# Procalcitonin as a Biomarker of Bacterial Infection in Sickle Cell Vaso-Occlusive Crisis

**DOI:** 10.4084/MJHID.2014.018

**Published:** 2014-02-17

**Authors:** Dilip Kumar Patel, Manoj Kumar Mohapatra, Ancil George Thomas, Siris Patel, Prasanta Purohit

**Affiliations:** 1 Department of Medicine. Veer Surendra Sai Medical College, Burla, Odisha, India; 2 Sickle Cell Clinic and Molecular Biology Laboratory, Veer Surendra Sai Medical College, Burla, Odisha, India

## Abstract

Sickle cell anaemia (SCA) patients with vaso-occlusive crisis (VOC) have signs of inflammation and it is often difficult to diagnose a bacterial infection in them. This study was undertaken to evaluate the role of serum procalcitonin (PCT) as a biomarker of bacterial infection in acute sickle cell vaso-occlusive crisis. Hundred homozygous SCA patients were studied at Sickle Cell Clinic and Molecular Biology Laboratory, V.S.S. Medical College, Burla, Odisha, India. All the patients were divided into three categories namely category-A (VOC/ACS with SIRS but without evidence of bacterial infection - 66 patients), category-B (VOC/ACS with SIRS and either proven or suspected bacterial infection - 24 patients) and category-C (SCA patients in steady state without VOC/ACS or SIRS - 10 patients). Complete blood count, C-reactive protein (CRP) estimation and PCT measurement were done in all the patients. There was no significant difference in TLC and CRP values between category-A and B. In category-A, the PCT level was <0.5 ng/mL in 83.3% and 0.5–2 ng/mL in 16.7% of cases. In category-B, all the patients had PCT value >0.5 ng/mL with 87.5% of patients having >2 ng/mL. In category-C, PCT value was <0.5 ng/mL. PCT had a high sensitivity (100%) and negative predictive value (100%) for bacterial infection at a cutoff value of 0.5 ng/mL; whereas the specificity is excellent at a cut-off value of 2 ng/mL. SCA patients with VOC/ACS and SIRS having a PCT level of <0.5 ng/mL have a low probability of bacterial infection whereas PCT value of >2 ng/mL is indicative of bacterial infection necessitating early antimicrobial therapy.

## Introduction

Sickle cell anaemia (SCA) is a genetic disorder resulting in the production of abnormal sickle haemoglobin. Various factors like hemolysis, chronic inflammation and endothelial dysfunction culminate in acute vaso-occlusion which is responsible for much of the morbidity observed in SCA patients.^1^,^2^ Vaso-occlusive crisis (VOC) is a common medical emergency in SCA patients necessitating hospitalization.^3^ Another complication namely infection is a major cause of morbidity and mortality in these patients. This is due to relative asplenic state and abnormal humoral immunity found in these patients.^4^,^5^ Moreover SCA patients with VOC may present with features of systemic inflammatory response syndrome (SIRS) like fever, tachycardia, tachypnea and raised leukocyte count without associated bacterial infection.^4^

Since untreated bacterial infection may result in serious complications with an unfavorable outcome, these patient are treated with antibiotics. However blind and over prescription of antibiotics in such a situation contributes to development of antimicrobial resistance, increases cost of management and exposes the patients to various side effects.^6^,^7^ Early diagnosis of bacterial infection in SCA patients presenting with VOC and SIRS is a challenge for emergency department physicians. Routine laboratory tests including total leukocytes count (TLC) and biomarkers such as C-reactive protein (CRP) have insufficient power and sensitivity for correctly identifying bacterial infection. Diagnosis of bacterial infection by microscopy or radiological examination may take 12–24 hours. Confirmatory microbial tests are unavailable for 24–48 hours.^8^ So to guide the use of antibiotics in the early hours of admission (within 6 hours) there is a need of a biomarker that could differentiate bacterial infection from nonspecific inflammation due to VOC.

Procalcitonin (PCT), the pro-hormone of calcitonin is normally produced by the thyroid gland in physiological condition. Its level increases thousand fold during acute bacterial infection.^9^,^10^ The level of PCT is found to correlate with the severity of bacterial infection and mortality.^11^–^14^ Several studies have reported that PCT can be used to distinguish systemic bacterial and fungal infection from viral and noninfectious causes of SIRS.^15^–^19^

Inherited haemoglobin disorders are highly prevalent in the state of Odisha in eastern India. In a cross-sectional prevalence study, the observed frequency of sickle cell gene was found to be 21% in this region.^20^ Advanced facility for diagnostic workup of microbial infections is unavailable in most of this region. For this reason, antibiotic overuse tends to be a large problem. Although PCT is a relatively expensive investigation (Indian Rupees 1000/- or 17 USD per test), the cost of empirical antibiotic therapy is higher. There are limited number of studies describing the usefulness of PCT in diagnosis of bacterial infection in patients with sickle cell disease (SCD) with conflicting results.^4^,^19^,^21^ Moreover, these studies have focused mostly SCD patients of Europe and USA with a different β-globin gene cluster haplotype and the results may not be applicable to Indian SCA patients with Asian-Indian haplotype. In view of this, we undertook this study with an aim to find the role of PCT as a biomarker of infection in Indian SCA patients.

## Materials and Methods

### Study design and patients

This prospective non-interventional observational mono-centric study was undertaken at the Sickle Cell Clinic and Molecular Biology Laboratory, Veer Surendra Sai Medical College Hospital, Burla in the state of Odisha, India, from October 2011 to September 2013. It is a referral centre for SCD patients located in the state of Odisha in eastern India. During the study period 152 SCA patients were admitted to the Department of Internal Medicine with VOC/ACS. After exclusion, 90 patients with VOC/ACS and SIRS were included in the study. Written informed consent was obtained from all the participants. Ten SCA patients in steady state without VOC/ACS attending to the Sickle Cell Clinic outdoor were taken as a control. The study was approved by the Institutional Ethical Committee.

### Definitions

VOC was defined as an acute painful event that required oral/injectable analgesics and that lasted for at least 4 hours when no other cause could explain the symptom.^22^,^23^ ACS was defined on the basis of the finding of a new pulmonary infiltrate involving at least one complete lung segment that was consistent with the presence of alveolar consolidation, but excluding atelectasis. In addition, the patients had to have chest pain, a temperature of more than 38.5°C, tachypnea, wheezing, or cough.^24^ SIRS was diagnosed in a patient with any two of the four clinical criteria namely hypothermia (<36^0^C), fever (>38^0^C), tachycardia (>90 beats/minute); and tachypnea (>20 breaths/minute).^25^

Bacterial infection was categorized into ‘proven bacterial infection” and “suspected bacterial infection”. Proven bacterial infection was defined when a causative bacterium could be identified by microscopy or culture of sputum, blood, urine and body fluid, supplemented with supportive history, clinical signs and symptoms. Suspected bacterial infection was defined when a causative bacterium could not be identified by microscopy or culture of sputum, blood, urine and body fluid, but clinical signs and symptoms were in concordance with radiological findings.^6^ For instance, a history of upper abdominal pain and fever with Murphy sign positive and ultrasound abdomen showing gallstones, pericholecystic fluid, gallbladder wall thickening (>4 mm) and sonographic Murphy sign was diagnosed as a case of suspected bacterial infection of gallbladder. When there were no supportive history, clinical sign and symptoms and a causative bacterium could not be identified by microscopy or culture of sputum, blood, urine or body fluid then the patient was categorized as a case of SCA with VOC/ACS and SIRS without proven or suspected bacterial infection.

### Exclusion criteria

Patients with the following criteria were excluded from the study: (a) Patients with other sickle cell syndromes such as HbSβ-thalassemia, HbSE, HbSC, HbSD-Punjab and others; (b) children under 14 years and adults above 60 years of age; (c) Patients who were a part of special program/trial that may have affected their clinical/haematological status; (d) Patients who could not be followed-up; (e) Patients who received antibiotics treatment prior to attending our facility; (f) Patients positive for malarial infection; (g) Patients who refused to participate in the study;

### Categorization of patients

Final diagnosis and categorization of the SCA patients was made at discharge using all of the available data including clinical, pathological, microbiological and radiological findings. Basing upon these 100 SCA patients were conveniently divided into three categories:

Category-A. VOC/ACS and SIRS without proven or suspected bacterial infection. All these cases were admitted to the indoors for a period varying from 3–7 days (median, 5 days). Category-B. VOC/ACS and SIRS with either proven or suspected bacterial infection. All these cases were admitted to the indoors for a period varying from 5–11 days (median, 7 days).

Category-C. SCA patients in steady state without VOC/ACS or SIRS attending to the Sickle Cell Clinic outdoor for routine checkup.

### Laboratory investigation

A sickling slide test and alkaline agarose gel haemoglobin electrophoresis (pH-8.6) were carried out as the initial screening procedures and those samples found positive in these two results were subjected to cation exchange high-performance liquid chromatography (CE-HPLC) using the VARIANT II Haemoglobin testing system; Bio-Rad Laboratories, Hercules, CA, USA as per the manufacturer’s guideline. Confirmation of SCA [codon 6 β(GAG>GTG) mutation] was done by amplification refractory mutation system-polymerase chain reaction (ARMS-PCR) using established protocols.^26^

A complete blood count (CBC) was carried out on an automated haematology analyzer (Sysmex KX-21; Sysmex Corporation, Kobe, Japan). CRP was done by latex turbidometry (SPINREACT, S.A./S.A.U. Ctra.Santa Coloma, 7 E-17176 SANT ESTEVE DE BAS (GI) Spain). Since the area under our study was a malaria endemic area, all the patients were screened for the same using peripheral smear by microscopy orImmunochromatographic test (ICT). Relevant investigations like plain radiography, sputum examination including microscopy and culture, culture of exudates and blood, ultrasonography were done as and when required. Urine examination including microscopy and culture was done for all the cases.

PCT was estimated semi-quantitatively within 6 hours of admission by using the BRAHMS PCT-Q kit (BRAHMS Aktiengesellschaft Neuendorfstrasse 25 D-16761 Hennigsdorf, Germany). As per the manufacturer’s guideline the semi-quantitative kit was able to detect PCT in the range of <0.5 ng/mL, 0.5–2.0 ng/mL, 2.0–10.0 ng/mL and ≥10 ng/mL.

### Follow-up

Following discharge from the hospital, each patient was followed up at the end of one week and one month to determine the course and fate of the illness. This was done at the outpatient department during the regular follow up sessions. Those who could not attend the same were followed up by telephonic conversation. At each visit during follow-up, detailed clinical, relevant microbiological and radiological investigations were performed in all the cases.

### Statistical analysis

Statistical analysis was done using GraphPad InStat Version 3.00 for Windows. For comparison between groups, the Mann-Whitney U test or Chi-square test were used as appropriate. TLC and CRP values in different PCT ranges were compared with Tukey-Kramer multiple comparison tests in patients with category-A and B. The diagnostic performance of PCT was reported as sensitivity, specificity, positive and negative predictive value for bacterial infection. *p* value of <0.05 was considered to be statistically significant.

## Result

### Patients

**Figure 1:**
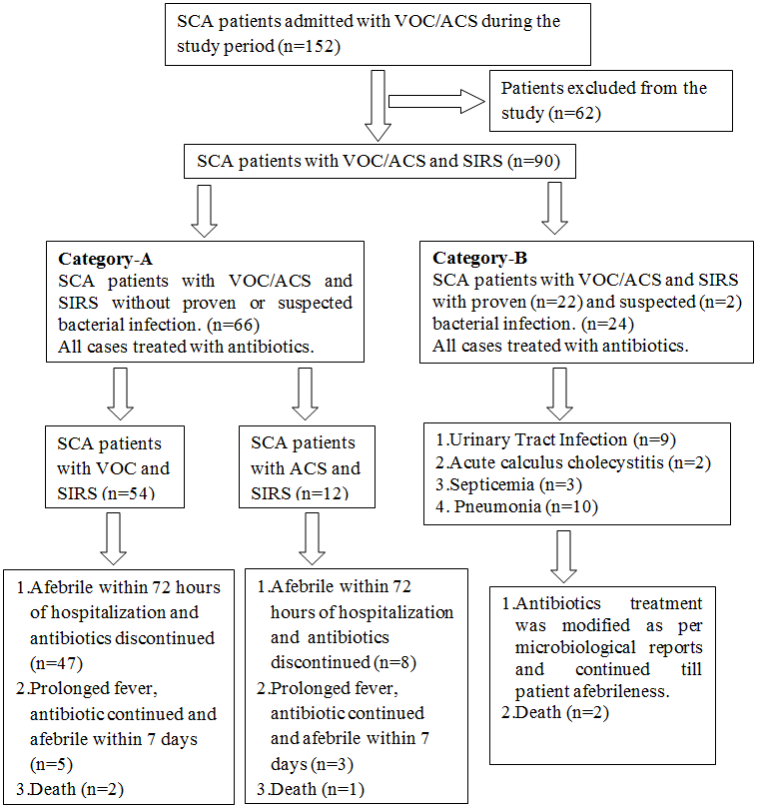
Recruitment procedure, categorization, treatment and outcome of sickle cell anaemia patients.

The study included 100 patients of SCA in the three categories of A, B and C, of whom, 52 patients were males. Category-A had 66 patients (VOC in 54, and ACS in 12 patients). Category-B included 24 patients of whom 22 had proven bacterial infection and rest 2 patients had suspected bacterial infection. Category-C had 10 patients of SCA in steady state without VOC/ACS or SIRS. The mean age of the study participants in category-A, B and C were 24.12±3.0, 23.04±5.04 and 26.3±2.2 years respectively. There was no difference in the age and sex distribution of cases in three categories. The recruitment procedure, categorization, treatment and outcome of SCA patients in the study are presented in a flowchart (Figure 1).

Of the 22 patients with proven bacterial infection, nine had urinary tract infection (*Escherichia coli* was isolated in seven cases, and two cases had *Klebsiella oxytoca*). Three patients of septicemia were diagnosed by positive blood culture (*Pseudomonas aeruginosa* was isolated in two cases, and one case had *Escherichia coli* infection). The rest ten patients in category-B had community acquired pneumonia (CAP) diagnosed radiologically. In these cases *Streptococcus pneumoniae* was isolated in five cases from sputum culture, three cases from blood culture and two cases from pleural fluid examination. Two patients with suspected bacterial infection had acute calculous cholecystitis diagnosed by clinical and ultrasonographic criteria. In these two patients blood culture was negative.

### Clinical parameters

Temperature and heart rate were significantly lower in category-C compared to both the category-A and B (*p*<0.001). Both these parameters were found to be significantly high in category-B compared to category-A (*p*<0.01).

### Laboratory findings

TLC and CRP were significantly lower in category-C compared to both the category-A and B (*p*<0.001). But there was no difference in values of these parameters between category-A and B. The baseline investigations and other parameters are provided in table 1. In category-A the PCT level was <0.5 ng/mL in 55 cases (83.3%) and 0.5–2 ng/mL in 11 (16.7%) cases. In category-B all the cases had PCT value >0.5 ng/mL. PCT level in various patients group in category-B is depicted in table 2. All the patients in category-C had PCT value of <0.5 ng/mL. The PCT value differed significantly (χ2, 89.17; *p* <0.0001) in the three categories of SCA patients.

**Figure 2:**
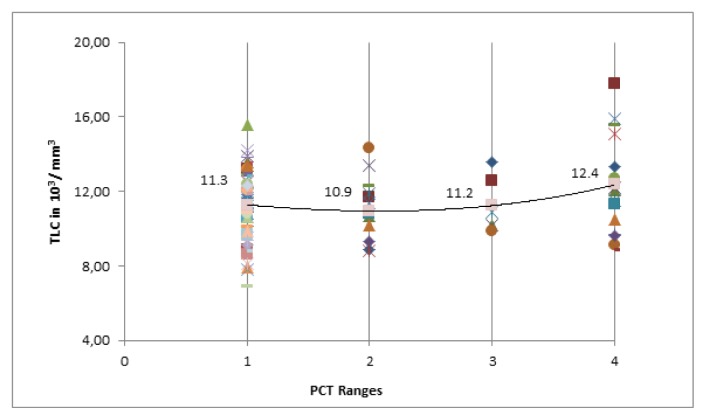
Comparison of TLC in four ranges of PCT in category A and B. PCT ranges 1, 2, 3 and 4 in graph represent <0.5, 0.5–2, 2–10 and ≥10 ng/mL ranges of PCT respectively. There was no significant difference in TLC level in four ranges of PCT (*p*>0.05). The increase in TLC level was inconsistent in patients without infection to with infection as increase in the PCT level. PCT-Procalcitonin; TLC-Total leucocyte count.

**Figure 3:**
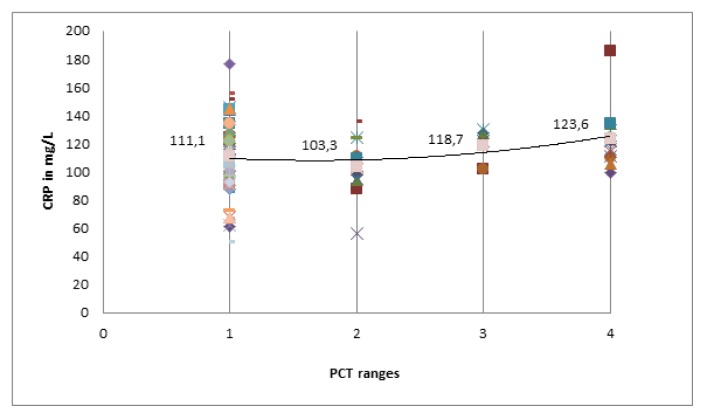
Comparison of CRP value in four ranges of PCT in category-A and B. PCT ranges 1, 2, 3 and 4 in graphs represent <0.5, 0.5–2, 2–10 and ≥10 ng/mL ranges of PCT respectively. There was no significant difference in CRP level in four ranges of PCT (*p*>0.05). The increase in CRP level was inconsistent in patients without infection to with infection as increase in the PCT level. PCT-Procalcitonin; CRP-C-Reactive protein.

A total of 14 patients (both in category-A and B) had a PCT value of 0.5–2 ng/mL of which majority belonged to category-A (78.5%). The TLC and CRP values were similar (*p*>0.05) when compared in four different ranges of PCT (< 0.5 ng/mL, 0.5–2.0 ng/mL, 2.0–10.0 ng/mL and ≥10 ng/mL) in category-A and B (Figure 2 and 3). PCT was evaluated as a potential biomarker of bacterial infection at various cut-off points. It was found that the test had a high sensitivity (100%) and negative predictive value (100%) at a cut-off value of 0.5 ng/mL; whereas the specificity is excellent at a cutoff value of 2 ng/mL (Table 3).

### Treatment and outcome

Empirical antibiotic treatment with 3^rd^ generation cephalosporin (ceftriaxone) was initiated in all 66 patients of category-A. 55 patients became afebrile in 3^rd^ day of hospital admission and had no evidences of bacterial infection for which antibiotic treatment was discontinued. In the rest, antibiotic was continued in view of persistent fever even though there was no documented bacterial infection. There were 3 deaths in this category; two cases died due to suspected pulmonary embolism on 4^th^ and 5^th^ day respectively and one case with ACS died on 5^th^ day of admission to the hospital.

In category-B, suitable empirical antibiotic treatment was started in all the patients. The antibiotic regimen was changed following the availability of a microbiological report. Two patients of acute calculous cholecystitis were treated with 3^rd^ generation cephalosporin (ceftriaxone) and metronidazole for a period of seven days; subsequently they were referred to Department of Surgery for cholecystectomy. In category-B, there were two deaths (one case had pneumonia with multiorgan dysfunction syndrome (MODS) who died on 4^th^ day and another case of septicemia died on 5^th^ day of hospital admission).

### Follow-up

All the 63 patients of category-A were clinically normal at the end of follow-up. In category-B, all the 9 patients with urinary tract infection were asymptomatic and afebrile at follow-up and urine examination was normal. Two patients of acute calculous cholecystitis underwent cholecystectomy in the second week and were better by symptoms at the end of one month. Both the patients of septicemia were afebrile and well. Nine patients of pneumonia improved clinically and radiologically at the end of follow-up and all except one had complete radiological resolution.

## Discussion

Diagnosis of bacterial infection is challenging because of the ambiguous clinical sign and limitation of microbial techniques.^27^ The causative microorganism cannot be detected in up to 80% of patients with suspected blood stream infections.^14^,^28^ Because of these facts there is a lack of a gold standard for invasive bacterial infection.^19^ Because of these diagnostic uncertainty several studies have analyzed the role of surrogate biomarkers like PCT to estimate the likelihood for presence of a bacterial infection in various clinical situations.^27^

It is interesting to learn that PCT belongs to the family of calcitonin gene related peptides and forms a functional entity during infection and inflammation.^29^ Multiple studies have demonstrated that serum levels of PCT are markedly increased in humans with sepsis, severe infection and severe inflammation.^10^ The serum values of PCT has been found to correlate with the severity of infection. Moreover, it has been demonstrated that PCT is a harmful biomarker and a therapeutic target for bacterial infection in humans. PCT has been found to be more useful and superior to other biomarkers of inflammation like CRP and TLC.^14^,^30^,^31^ As a diagnostic marker, PCT has several advantages over CRP because it increases in an earlier stage of infection followed by rapid decline when the infection is controlled by immune system or antibiotic treatment. Further unlikely CRP, production of PCT is not attenuated by steroidal and non-steroidal anti-inflammatory drugs.^9^ In the present study we validated the role of PCT for triage for selective and early institution (within 6 hours of admission) of antimicrobials in SCA patients presenting with VOC/ACS and SIRS.

In this study, clinical parameters like temperature and heart rate were significantly high in patients with proven or suspected bacterial infection. However laboratory parameters of inflammation like TLC and CRP were similar in both category-A and B. Reliance on these markers would have led to overtreatment, undesired exposure to antibiotics with antecedent consequences.^4^ In the study by Stojanovic et al.,^19^ the TLC could not differentiate SCA patients with invasive bacterial infection from those without it. Similarly, CRP level was non discriminative in both the groups of patients. In another study, the CRP level was found to be significantly high in SCA patients with VOC associated with fever in comparison to sickle vaso-occlusive crisis patients without fever. However this study is less informative as the authors did not take into account the possibility of bacterial infection as a cause of fever in SCA patients.^21^

In the present study, the PCT was significantly high in category-A (SCA patients with VOC/ACS and SIRS without bacterial infection) and category-B (SCA patients with VOC and SIRS with either proven or suspected bacterial infection) in comparison to category-C (SCA patients without VOC/ACS or bacterial infection). In a study of 24 cases of SCA, Scott et al.,^4^ reported that all the 5 patients with documented bacterial infection at presentation had PCT ≥2 ng/mL. They estimated the PCT in semi-quantitative kit method similar to that of ours. In another recent study PCT level was estimated by an ultrasensitive method in 6 SCA patients with fever, VOC and documented invasive bacterial infection. The mean PCT value was 1.98 μg/L with a range from 0.10 to 5.99 μg/L. Four of these patients had a PCT value of ≥1 μg/L whereas two had <1 μg/L (0.09 and 0.10 μg/L, respectively). The authors concluded that high level PCT (≥1 μg/L) indicate invasive bacterial infection.^19^

The probability of bacterial infection was very low in SCA patients with a PCT value of <0.5 ng/ml. At this level serum PCT had a negative predictive value (100%) in excluding bacterial infection. In the study by Scott et al.,^4^ a serum PCT level <2 ng/mL had a strong negative predictive value in excluding bacterial infection. However, Stojanovic et al.,^19^ concluded that a single low PCT level without follow-up measurement does not rule out invasive bacterial infection as 33% of their patients had low PCT value. We found that all but 3 of the patients with proven and suspected bacterial infection (n=24) had a PCT value of >2 ng/mL. At this level PCT had 100% specificity and positive predictive value (100%) for presence of bacterial infection in SCA with VOC and SIRS. In view of high probability of bacterial infection in this group antimicrobial therapy may be continued until clinical recovery. In the Parisian study, PCT was estimated by an ultra sensitive ELISA method, and both the specificity and positive predictive value were 100% at PCT value of ≥1 μg/L.^19^

**Figure 4:**
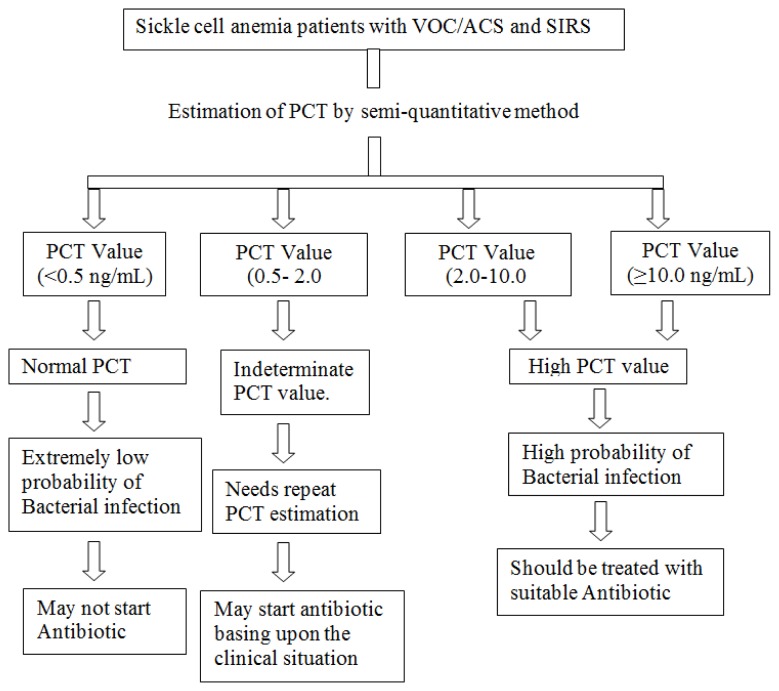
Clinical algorithm for guidance of antibiotic therapy in sickle cell anaemia patients with VOC/ACS and SIRS.

In the present study, 14 cases (11/66 cases from category-A and 3/24 cases from category-B) had a PCT value in the range of 0.5–2.0 ng/mL. This was an indeterminate value and there was overlap of both category-A and B patients. In view of this it would be justifiable to repeat this test and antimicrobial treatment may be instituted basing upon the clinical condition of the patient. Majority of the patients (62.5%) in the bacterial infection category had a PCT level >10.0 ng/mL. However in view of small number of deaths in our study both in category-A and B, we could not correlate the high PCT level with adverse outcome like death. Basing upon the result of PCT value in our patients we have proposed a clinical algorithm for guidance of anti-microbial treatment (Figure 4).

PCT has not been studied as a biomarker for prognostic assessment of bacterial infection in SCA patients with VOC and SIRS. Several studies have been undertaken in lower respiratory tract infection (LRTI), community acquired pneumonia (CAP) and sepsis to study the prognostic implication of PCT.^27^ In the ICU setting elevated PCT level was an independent predictor for 90 days all cause mortality in patients with sepsis. In this situation high CRP level and TLC did not predict mortality.^32^ In CAP, PCT seems to be a useful diagnostic marker but is not an ideal prognostic tool.^27^

There are some limitations of this observational study. We evaluated the role of PCT in only one tertiary hospital and the numbers of SCA patients with suspected or proven bacterial infection were modest. Therefore the clinical algorithm proposed by us needs prospective evaluation and external validation in multicentre study with more number of patients. Microbial culture is insensitive and there is no gold standard for bacterial infection. So some SCA patients with bacterial infection with negative microbial culture may have been included in category-A. We employed a semi quantitative immunochromatographic method for estimation of PCT. An ultra-sensitive ELISA test if employed could have given more credibility. However, we did not use this ultra-sensitive method in view of financial constrain.

## Conclusions

From the above findings it can be presumed that SCA patients with VOC/ACS with SIRS presenting to the emergency department with a PCT level of <0.5ng/mL have a low probability of bacterial infection. These patients may not be administered antibiotics and can be managed with supportive therapy. Those patients with PCT >2 ng/mL have a high probability of bacterial infection and it is prudent to initiate antibacterial therapy without waiting for results of bacteriological tests. In patients with indeterminate PCT value of 0.5 to 2ng/mL, there is a need for repeat PCT estimation. Empirical antibiotic therapy may be initiated in these patients awaiting the availability of microbiological report. In developing countries with limited resources, PCT enhanced triage will support the choice of starting antibiotic therapy, reduce the overall cost of patient management and shorten unnecessary hospital stay while achieving similar quality of life and patient outcome.

## Figures and Tables

**Table 1:** General characteristics and laboratory findings of sickle cell anaemia patients in three categories.

	Category- A (n=66)	Category- B (n=24)	Category- C (n=10)	*p* Value
	
Age	24.12 ± 3.0	23.04 ± 5.04	26.3 ± 2.2	0.058
Sex				
Male	36	10	6	
Female	30	14	4	>0.05
Heart Rate (/min)	104.6 ±8.4*#	111.5 ± 8.7*	78.9 ± 6.1	<0.0001
Systolic BP (mm of Hg)	116.5 ± 7.3	112.5±13.4	118.2 ± 7.5	0.1289
Diastolic BP (mm of Hg)	71.7 ± 8.5	70.7 ± 9.1	77.4 ± 6.4	0.1015
Temperature (®F)	100.0 ± 1.3*#	101.3 ± 1.6*	98.6 ± 1.2	<0.0001
Hospitalization (Days in Median)	5*	7*	0	<0.0001
TLC (×10^3^/mm )	11.2 ± 1.8*	11.8 ± 2.4*	8.3 ± 1.4	<0.0001
Hemoglobin (gm/dL)	8.04 ± 1.01	7.8 ± 1.4	8.72 ± 0.93	0.0828
CRP (mg/L)	110.3 ± 25.5*	118.6±19.6*	8.0 ± 3.06	<0.0001
Mortality (%)	4.54	8.33	0	>0.05

Category-A. SCA patients with VOC/ACS and SIRS without proven or suspected bacterial infection; Category-B. SCA patients with VOC/ACS and SIRS without proven or suspected bacterial infection; Category-C. Patients in steady state without VOC/ACS or SIRS.

* *p*<0.001 compared with Category C;

# p<0.01 compared with Category B.

BP; Blood Pressure; TLC: Total Leucocyte count; CRP: C-Reactive protein.

**Table 2:** PCT level in sickle cell anaemia patients with various bacterial infections in category-B.

Types of bacterial infection	<0.5 ng/mL	0.5–2.0 ng/mL	2.0–10.0 ng/mL	≥10 ng/mL
Urinary Tract Infection (n=9)	0	3	3	3
Acute Calculus Cholecystitis (n=2)	0	0	1	1
Septicaemia (n=3)	0	0	0	3
Pneumonia (n=10)	0	0	2	8

Total	0	3	6	15

**Table 3:** Descriptive statistics of the accuracy of procalcitonin (PCT) as a biomarker for bacterial infections in sickle cell anaemia patients using various cut-off points.

Sickle cell anaemia cases	Procalcitonin Cut-off points		
	0.5 ng/mL	2.0ng/mL	10.0 ng/mL
Sensitivity	100.00 % (85.62 % to 100.00 %)	87.50 % (67.61% to 97.20 %)	62.50 % (40.60 % to 81.16 %)
Specificity	85.53% (75.57 % to 92.54 %)	**100.00 %** (95.21% to 100%)	100.00 % (95.21 % to 100.00 %)
Positive Predictive Value	68.57 % (50.71 % to 83.13%)	100.00 % (83.75% to 100.00 %)	100.00 % (78.03 % to 100.00 %)
Negative Predictive Value	**100.00 %** (94.43 % to 100.00 %)	96.20 % (89.29 % to 99.17 %)	88.46 % (80.84 % to 95.03 %)

Data is shown with 95% Confidence Interval.
